# Acute physiological responses during repeated sprint training with maximum end-expiratory breath-holds

**DOI:** 10.1007/s00421-025-06030-7

**Published:** 2025-11-03

**Authors:** Antoine Raberin, Tom Citherlet, Marco Carletta, Niccolò Franchi, Giorgio Manferdelli, Grégoire P. Millet

**Affiliations:** Institute of Sport Sciences, University of Lausanne, Lausanne, 1015 Switzerland

**Keywords:** Hypoxia, Hypercapnia, Voluntary hypoventilation, Apnea

## Abstract

**Purpose:**

Repeated sprint training in hypoxia (RSH) performed with voluntary hypoventilation at low lung volumes (RSH-VHL) has been proposed as an alternative to RSH. Recently, repeated sprint training combined with end-expiratory breath hold until the breaking point (RSH-UBP) has been suggested as a more effective stimulus. The aim of this study was to compare the physiological responses of repeated sprint training in normoxia (RSN), RSH-VHL and RSH-UBP. We hypothesized that RSH-UBP would induce greater hypercapnic and hypoxemic stress compared to the other conditions and therefore greater physiological responses.

**Methods:**

Ten healthy active men participated in three repeated sprint exercise sessions on ergocycle. Pulse oxygen saturation (SpO_2_), end-tidal CO_2_ pressure (P_ET_CO_2_), muscle oxygenation, cardiac hemodynamics, and total work were continuously monitored throughout the protocol.

**Results:**

Time spent below 96% SpO_2_ was higher in both RSH-VHL and RSH-UBP than in RSN (82 ± 66 s and 74 ± 57 s vs. 11 ± 14 s, *P* = 0.039). Contradictory, P_ET_CO_2_ was higher in RSH-VHL and RSH-UBP than in RSN (33.6 ± 4.2 and 32.9 ± 3.5 vs. 27.8 ± 4.2 mmHg, *p* < 0.001). Total work was lower in RSH-UBP than in RSH-VHL and RSN (33.1 ± 5.8 vs. 46.5 ± 5.7 and 47.4 ± 6.0 kJ, *p* < 0.001) despite a higher mean power in RSH-UBP. Amplitude in the muscle deoxygenation-reoxygenation cycle was lower during RSH-UBP (*p* < 0.045). There was no difference in stroke volume or cardiac output between the three conditions.

**Conclusion:**

Overall, RSH-VHL emerged as the most effective condition for inducing large hypercapnic and hypoxemic stimuli while maintaining the training load during repeated sprint training, whereas RSH-UBP succeeded in inducing similar hypercapnic and hypoxic stress with shorter sprint durations.

## Introduction

In intermittent sports, such as team, combat, or racket sports, repeated sprint ability (RSA), i.e., the capacity to repeat all-out efforts with incomplete recovery, is a key determinant of performance (Bishop et al. 2011). In the last decade, repeated sprint training in hypoxia (RSH) emerged as an effective method for enhancing RSA, inducing larger RSA improvement than the same training conducted in normoxia (RSN) (Brocherie et al. 2017; Millet et al. 2019; Faiss et al. 2024). RSH peripheral adaptations suspected to underlie its efficiency are phenotypic changes favoring fast-twitch (FT) fibers and increase in myoglobin content mediated by hypoxia inducible factors (Faiss et al. 2013, 2024; Nava et al. 2022), the hypoxia-induced compensatory peripheral vasodilation (Casey et al. 2010; Casey and Joyner 2012; Faiss et al. 2024), and behavioral change of FT fibers, which benefit from the increased blood perfusion (Faiss et al. 2013, 2024).

Since access to the hypoxic setup requested for RSH may not be available for every athlete, an alternative method called repeated sprint training in hypoxia induced by voluntary hypoventilation at low lung volume (RSH-VHL) was developed (Trincat et al. 2017; Woorons et al. 2017). Voluntary hypoventilation at low lung volume (VHL) is a specific breathing technique which involve a voluntary modification of the breathing pattern. To practice VHL, individuals performed an exhalation down to functional residual capacity and hold their breath at this specific lung volume (i.e., end expiratory breath hold). At the end of the breath hold, individuals perform a forced expiration down to residual volume before complete inhalation. This ventilatory pattern can be repeated during an entire exercise bout of several minutes (Woorons et al. 2010) or only during short all-out sprints (Woorons et al. 2017), with normal breathing allowed during inter-sprints recovery. Exercise with VHL induces larger arterial and muscle desaturation than exercise performed with normal breathing (Woorons et al. 2010, 2017). Moreover, mean power output is not altered during RSH-VHL session (Woorons et al. 2017). RSH-VHL training interventions have reported greater benefits than RSN, with a higher number of sprints to exhaustion (Trincat et al. 2017; Fornasier-Santos et al. 2018; Ait Ali Braham et al. 2024), a higher mean performance (Woorons et al. 2019b) and a lower performance decrement during RSA (Lapointe et al. 2020; Brocherie et al. 2023).

However, VHL acutely generates relative hypercapnia and acidosis, when compared to normal breathing (Woorons et al. 2010, 2023). It also induces an increase in stroke volume (SV) during recovery periods (Woorons et al. 2011, 2017), which has been attributed to the large and brief inspiration occurring after the breath hold, that enhances the venous return to the heart (Woorons et al. 2021b).

Recently, the possibility of exercising with end-expiratory breath-holding maintained up to the breaking point (UBP) has emerged, with larger hypoxemic and relative hypercapnic stimuli during high-intensity interval training (Woorons et al. 2020, 2021a, b, 2023). This breathing pattern induced higher values in SV (Woorons et al. 2021b), muscle deoxygenation (Woorons et al. 2021a, 2023), and pulse oxygen desaturation (Woorons et al. 2021a, b, 2023) than normal breathing during exercises performed at 60 to 150% of maximal aerobic power/speed. However, to our knowledge, repeated sprint exercise with end-expiratory breath-hold until the breaking point (RSH-UBP) has never been compared to RSH-VHL with fixed sprint durations or distances.

Therefore, this study aimed to compare the physiological responses of RSN, RSH-VHL and RSH-UBP. Due to the putative higher hypoxic stimulus, relative hypercapnic stimulus and change in intrathoracic pressure during ventilatory maneuver in RSH-UBP when compared to RSN and RSH-VHL, we hypothesized that RSH-UPB would lead to greater SV changes and muscle deoxygenation/reoxygenation during repeated sprints.

## Methods

### Participants

Ten healthy active men were included in this study. They were non-smokers, physically active (≥ 2 h of training/week) and did not suffer from any known cardiovascular, metabolic, or pulmonary disease. To avoid any influence from prior experience user of the VHL method, swimmers or individuals with prior breath-holding experience were excluded. Approval for this study was obtained from the institutional ethical committee (CER-VD 138/15) and was conformed with the Declaration of Helsinki. All participants provided written informed consent prior to participation.

### Protocol

**Fig. 1:**
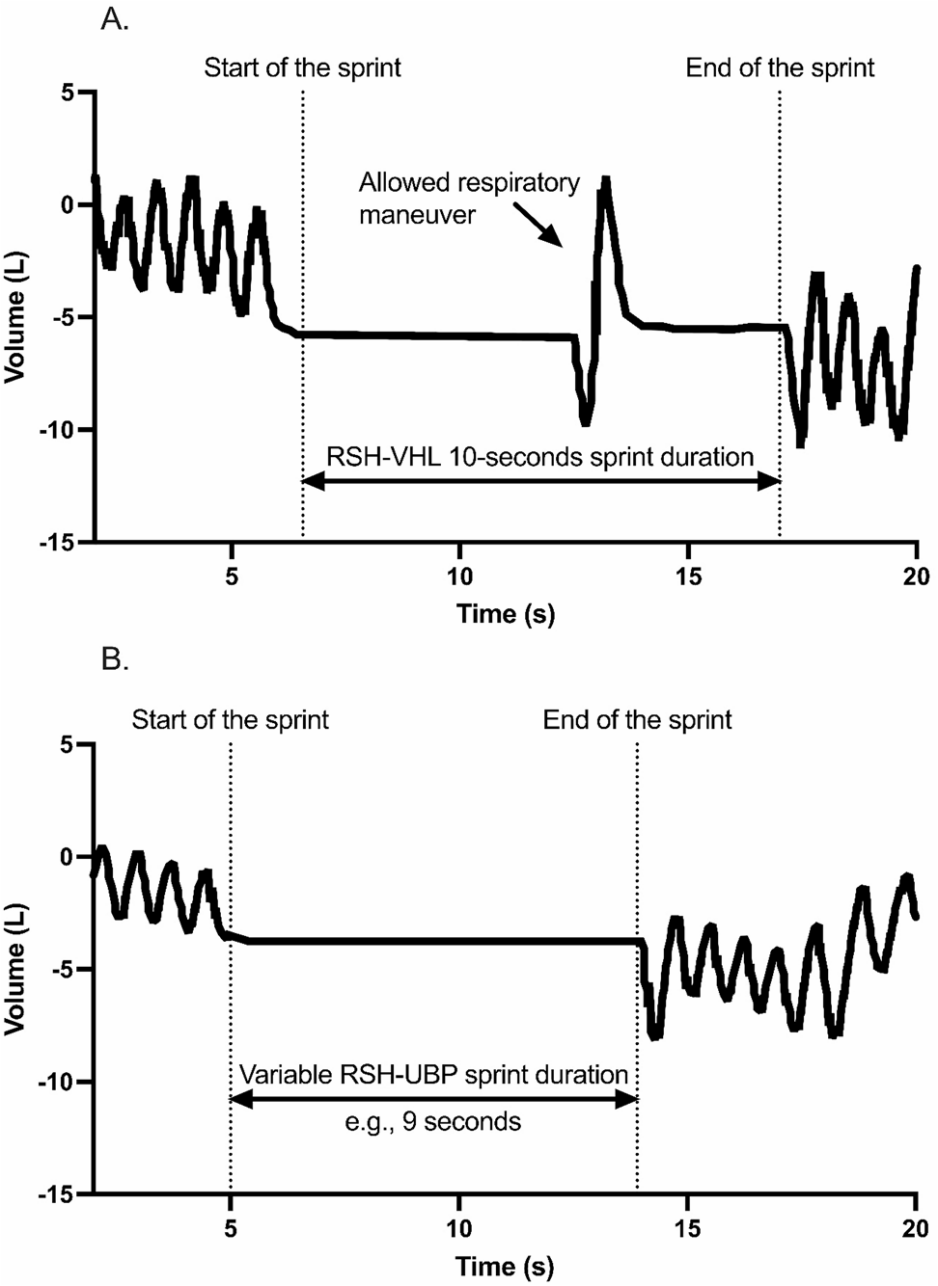
Representative tidal volume over time signal during a sprint performed in (**A**) voluntary hypoventilation at low lung volume- (RSH-VHL) or in (**B**) end-expiratory breath-hold until the breaking point- (RSH-UBP)

The experimental protocol consisted of three sessions of repeated sprint with three different breathing patterns and a minimum of 48 h between each session. One of the sessions took place with unrestrictive breathing (RSN). Another one with voluntary hypoventilation at low lung volume (RSH-VHL); i.e., participants started their sprint in VHL and were allowed to quickly expire down to residual volume inhale and come back to functional residual capacity once during each sprint if they felt the need to breath (Fig. 1A). During the third session, participants performed sprints with end-expiratory breath-hold until the breaking point (RSH-UBP); i.e., they sprinted at functional residual capacity as long as they can (Fig. 1B). The order of the sessions was randomized. The sprint duration was 10 s for RSN and RSH-VHL while it depends on the apnea time for RSH-UBP. The exercise-to-rest ratio was 1:2 in all sessions. The 10-second sprint was chosen because it had previously been used for intense UBP bouts at 150% of maximal power output (Woorons et al. 2021b) and because intensity does not seem to alter apnea duration during UBP (Woorons et al. 2021a). A 10-second sprint duration combined with a 1:2 exercise-to-rest ratio is also considered ideal for balancing oxidative and glycolytic energy system contributions, promoting optimal adaptations during RSH (Raberin et al. 2022, 2023; Faiss et al. 2024).

Each session consisted of 2 sets of 8 sprints, with 5 minutes of recovery at 20 W between sets. All sprints were performed on an ergocycle (Excalibur, Lode, Groningen, Netherlands) in ‘Wingate mode’ with a fixed resistance (torque factor of 0.8 N·m·kg⁻¹). Sprints were initiated from a rolling start, with participants targeting a pedaling frequency of 85 rpm at 20 W. The load between each sprint was set at 20 W.

Each session started with a familiarization and training on the breathing pattern of the session. Then the warm-up began with 5 min at 50 W followed by 5 min at 100 W. Participants finished the warm-up by performing two isolated sprints using the breathing pattern of the session. It was followed by 5 min at 20 W before the start of the repeated sprint bouts. For RSN and RSH-VHL, sprint and recovery duration were predetermined while for RSH-UBP, each inter-sprint recovery duration was continuously adapted to fit with the 1:2 exercise-to-rest ratio.

### Measurements

#### Hypoxemic and relative hypercapnic stimuli

Hypoxic and relative hypercapnic stimuli were quantified using pulse oxygen saturation (SpO_2_) and end-tidal CO_2_ pressure (P_ET_CO_2_), respectively, which were monitored throughout the entire session. SpO_2_ was measured by a pulse oximeter (Wristox, Nonin Medical Inc, Plymouth, MN, USA) with a sensor attached to participant’s earlobe. Before starting the recording, the earlobe was pre-warmed using vasodilator cream (Capsolin, SIT s.r.l., Mede, Italy). Hypoxic stimulus was measured as the time spent with SpO_2_ below 96%, as previously reported (Trincat et al. 2017). P_ET_CO_2_ was measured by a breath-by-breath metabolic analyzer (K5, COSMED, Rome, Italy) that was calibrated according to the manufacturer’s recommendations. The mean P_ET_CO_2_ during each set was used to characterize the relative hypercapnic stimulus.

#### Power output, sprint durations and total work

Sprint duration, as well as peak and mean power output (averaged across all sprints), were recorded by the cycle ergometer. Total work for each set was calculated as the product of sprint duration and mean power output during the set.

#### Blood gases

A capillary blood sample (70 µL) was collected from each participant’s earlobe at rest before the warmup and immediately after the final sprint. Arterialization of the capillary blood was achieved by applying a vasodilator cream (Capsolin; SIT s.r.l., Mede, Italy) prior to testing. Samples were analyzed immediately using a blood gas analyzer (Opti CCA TS-2, Anandic Medical System, Feuerthalen, Switzerland) to measure pH, arterial partial pressure of CO_2_ (PaCO_2_) and O_2_ (PaO_2_), and bicarbonate ion (HCO_3_⁻). Data are presented as the difference (Δ) between the sample at rest and the sample collected after the final sprint.

#### Muscle oxygenation

During the sessions, oxygenation of the *vastus lateralis* muscle was continuously monitored using a near-infrared spectroscopy (NIRS) system (Portamon, Artinis Medical Systems BV, Elst, The Netherlands). This NIRS technique has been extensively described and validated in the literature (Perrey and Ferrari 2018). Briefly, NIRS light-emitting diodes (wavelengths: 760 and 850 nm) enable the measurement of micromolar changes in oxygenated and deoxygenated hemoglobin ([O_2_Hb] and [HHb], respectively). The sum of the changes at 760 and 850 nm provides the micromolar change in total hemoglobin concentration ([tHb]), reflecting variations in regional blood volume. The ratio of [O_2_Hb] to [tHb] was used to calculate the tissue saturation index (TSI). A differential path length factor of 4 was applied in all tests, and muscle oxygenation was recorded at 50 Hz. A fourth-order low-pass zero-phase Butterworth filter (cutoff frequency, 0.2 Hz) was applied to the resampled signals to remove possible artifacts and smooth the pedaling-induced perturbations.

Optodes were placed on the right belly of the *vastus lateralis* muscle, midway between the greater trochanter and the lateral epicondyle of the femur. After initial placement, marks were made on the skin at the probe’s edges to confirm stability during the trial and facilitate exact re-placement in subsequent sessions. The probe was then securely fixed to the thigh using tape, a bandage, and the participant’s shorts, minimizing ambient light interference.

The maximum and minimum NIRS signal values were identified for each sprint and recovery period. Typically, maximum [O_2_Hb], [tHb], and TSI, along with minimum [HHb], occurred at the end of each recovery - start of each sprint. Conversely, minimum [O_2_Hb], [tHb], and TSI, as well as maximum [HHb], were recorded at the end of each sprint – start of each recovery. The differences in minimum and maximum concentrations between the beginning and end of each sprint were defined as the amplitude of variation (Δ[O_2_Hb], Δ[HHb], Δ[tHb], and ΔTSI).

#### Cardiac hemodynamics

Heart rate (HR) and SV were measured beat-by-beat using automated impedance cardiography (Physioflow Enduro; Manatec Biomedical, Paris, France) and averaged every second. The accuracy of this device for monitoring cardiac dynamics during exercise in healthy individuals has been previously validated against the direct Fick method (Richard et al. 2001), and it has also been employed in studies involving breath-hold training until the breaking point (Woorons et al. 2021b). Cardiac output (Q̇) was calculated as HR × SV.

To optimize the impedance signal, the skin was carefully shaved and cleaned, and electrodes were securely attached to the skin with tape. The device was calibrated before each test according to the manufacturer’s instructions. HR, SV, and Q̇ values were expressed as the average over the cumulative sprint period of a set, the recovery period of a set, or the entire set. Data points with a signal quality below 80% were excluded from analysis.

### Statistical analysis

To achieve a statistical power of 0.8 and a significance level of 0.05 for repeated-measures ANOVA, the a-priori calculated sample size was *n* = 10, based on prior research related to RSH-VHL (Woorons et al. 2021a, b, 2023; Imai et al. 2022).

Data were first tested for normality using Q-Q plots and skewness inspection. If normal distribution was confirmed, a one-way repeated-measures ANOVA was used for analysis. When the assumption of sphericity was violated, the Greenhouse-Geisser correction was applied. If normality was not confirmed, data were analyzed using Friedman’s ANOVA on ranks. When a significant ANOVA result was found, pairwise comparisons with Bonferroni adjustment were conducted to identify specific differences.

All statistical analyses were conducted using IBM SPSS Statistics (version 29.0, IBM, NY, USA). Results are expressed as mean ± standard deviation.

## Results

Ten men completed the entire protocol (age: 29.4 ± 11.4 years, height: 181 ± 6 cm, body mass: 76.3 ± 5.7 kg, body mass index: 23.1 ± 1.5 kg.m^− 1^, skinfold thickness at the NIRS probe site: 10.8 ± 6.1 mm).

**Fig. 2:**
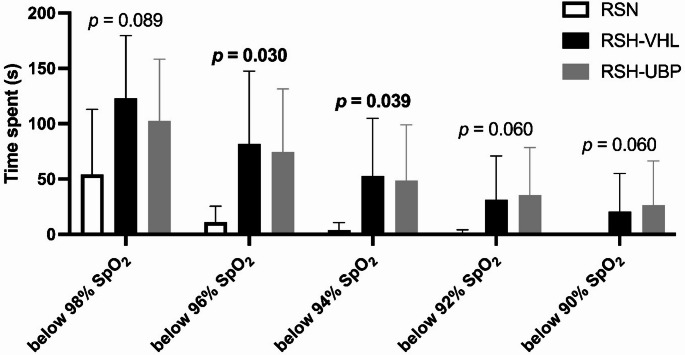
Time spent below pulse oxygen saturation (SpO_2_) thresholds during unrestrictive breathing- (RSN), voluntary hypoventilation at low lung volume- (RSH-VHL), and end-expiratory breath-hold until the breaking point- (RSH-UBP) sessions

**Fig. 3:**
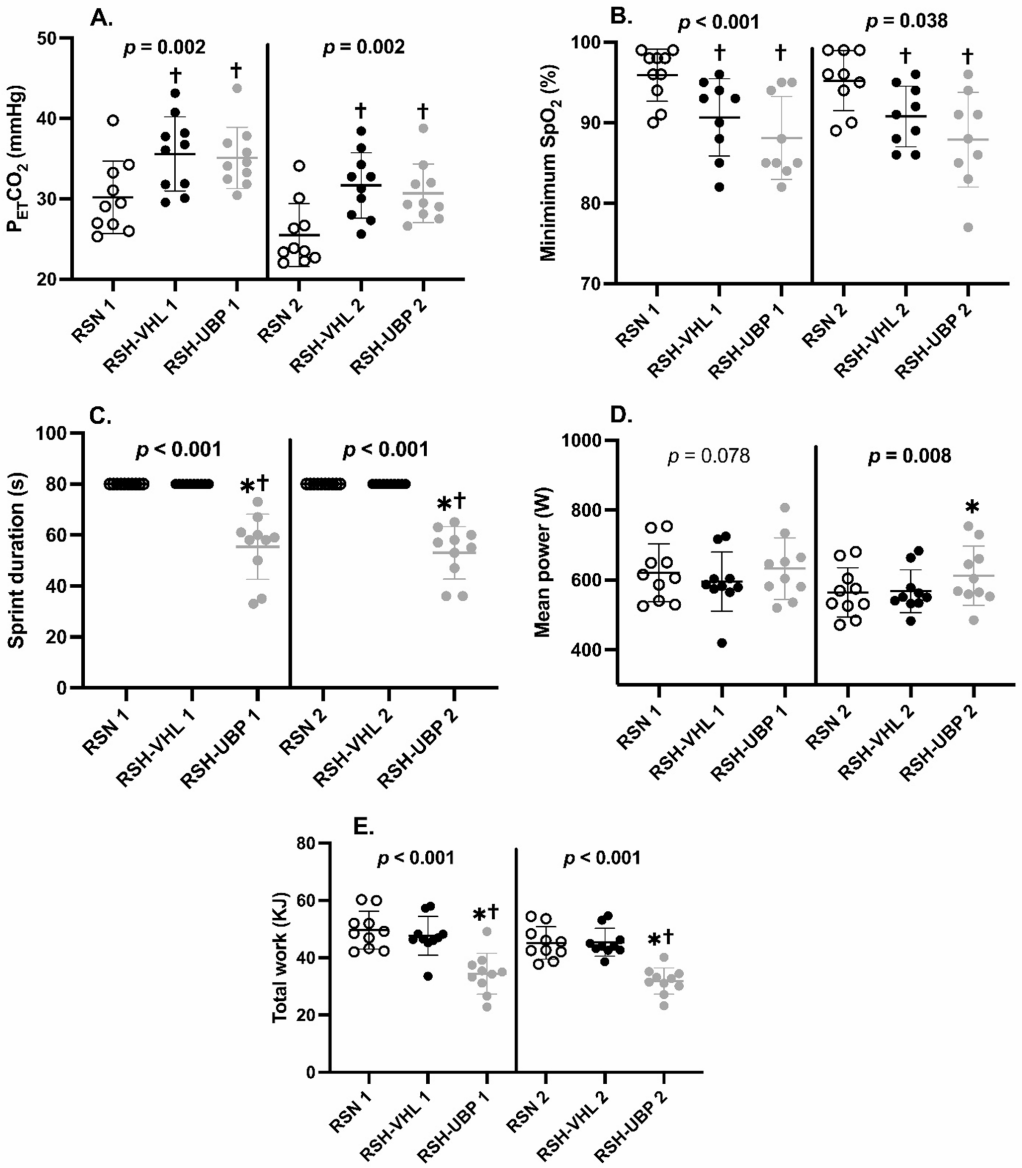
Relative hypercapnic and load stimuli during the first and second sets of sprints in unrestrictive breathing- (RSN), voluntary hypoventilation at low lung volume- (RSH-VHL), and end-expiratory breath-hold until the breaking point- (RSH-UBP) sessions. Panel A: End tidal partial pressure of CO_2_. Panel B: minimum SpO_2_. Panel C: sprint duration. Panel D: mean power output. Panel E: total work. *Different from RSH-VHL, ^†^different from RSN

There were significant differences among the three conditions in the time spent with SpO_2_ levels below 96% (K = 7.032, *p* = 0.030) and below 94% (K = 6.500, *p* = 0.039) (Fig. 2) and in minimum SpO_2_ during the first (F = 13.074, *p* < 0.001) and second set (F = 4.171, *p* = 0.038) (Fig. 3B). No significant differences were found for the time spent with SpO_2_ levels below 98%, 92%, and 90% (F = 4.839, *p* = 0.089; K = 5.630, *p* = 0.060; and K = 5.630, *p* = 0.060, respectively).

P_ET_CO_2_ differed significantly among the three conditions during both the first set (F = 8.524, *p* = 0.002) and the second set (K = 12.600, *p* = 0.002) (Fig. 3A). In both sets, P_ET_CO_2_ levels were higher during RSH-VHL and RSH-UBP conditions, compared to RSN.

**Fig. 4:**
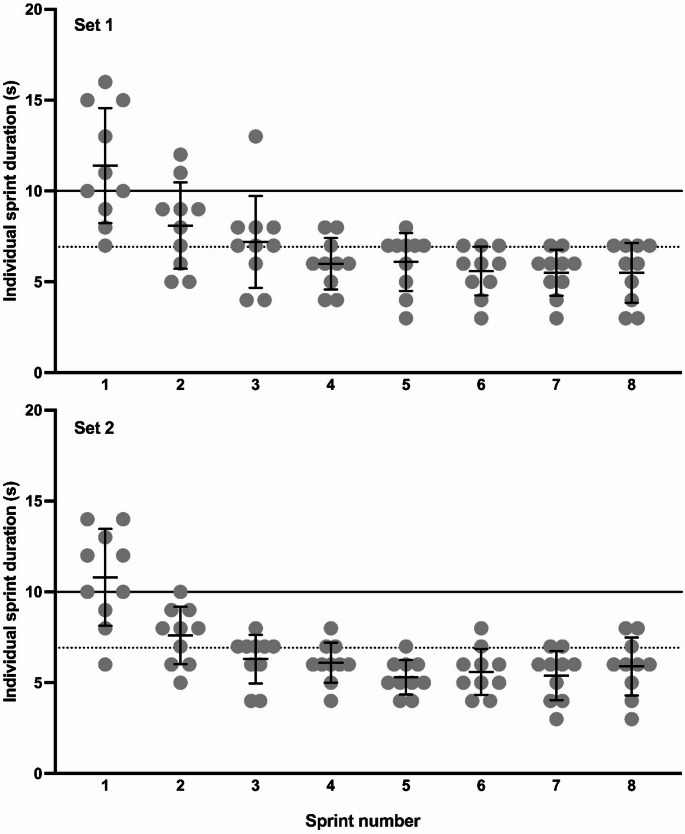
Individual sprint time during end-expiratory breath-hold until the breaking point- (RSH-UBP) in set 1 and set 2. The continuous line represents the 10 s duration used in the two other conditions. The dotted line is the average duration of every sprints

Mean power output differed significantly during the second set (F = 6.483, *p* = 0.008) but not in the first set (F = 3.014, *p* = 0.078) (Fig. 3D). Cumulative sprint time was lower in the RSH-UBP condition compared to the fixed sprint time (80 s) in the other two conditions for both the first (T = 6.086, *p* < 0.001) and the second sets (T = 8.302, *p* < 0.001) (Fig. 3C). Individual sprint time are reported in Fig. 4.

Total work differed significantly among the three conditions in both the first (F = 27.675, *p* < 0.001) and the second sets (F = 45.971, *p* < 0.001) (Fig. 3E). In both sets, total work produced was lower in the RSH-UBP condition, compared to RSH-VHL and RSN.

Changes in blood gases, pH, and bicarbonate (HCO_3_⁻) levels during the sessions are reported in Table 1. No significant differences were observed between the RSN, RSH-VHL, and RSH-UBP.

**Table 1:** Pre- to post-sprint session changes in blood gases, pH, and bicarbonate

Variable	RSN	RSH-VHL	RSH-UBP	Test statistic	*p*-value
Pre PO_2_ (mmHg)	75.6 ± 5.6	79.7 ± 12.0	75.8 ± 4.5	F = 0.466	0.545
Pre PCO_2_ (mmHg)	37.6 ± 3.2	38.5 ± 2.1	37.4 ± 2.2	F = 0.778	0.478
Pre pH	7.4 ± 0.02	7.43 ± 0.02	7.44 ± 0.01	F = 1.350	0.287
Pre HCO_3_^−^ (mmol.L^− 1^)	25.4 ± 1.3	25.2 ± 1.5	25.0 ± 1.5	F = 0.245	0.786
Post PO_2_ (mmHg)	81.2 ± 5.3	80.1 ± 2.5	81.8 ± 2.5	K = 2.000	0.368
Post PCO_2_ (mmHg)	30.4 ± 3.5	32.8 ± 2.7	32.4 ± 1.8	F = 2.512	0.117
Post pH	7.21 ± 0.06	7.25 ± 0.12	7.24 ± 0.07	F = 1.308	0.298
Post HCO_3_^−^ (mmol.L^− 1^)	12.2 ± 2.8	13.0 ± 1.9	13.6 ± 2.6	F = 2.256	0.137
ΔPO_2_ (mmHg)	−6.4 ± 3.8	−0.7 ± 10.6	−7.1 ± 2.9	K = 0.286	0.867
ΔPCO_2_ (mmHg)	7.2 ± 2.7	5.7 ± 3.1	4.9 ± 3.5	F = 1.269	0.311
ΔpH	0.23 ± 0.07	0.18 ± 0.10	0.20 ± 0.06	K = 4.222	0.121
ΔHCO_3_^−^ (mmol.L^− 1^)	13.2 ± 2.3	12.2 ± 1.9	11.4 ± 3.1	F = 2.591	0.106

*PO*_*2*_ partial pressure of oxygen, *PCO*_*2*_ partial pressure of carbon dioxide, *pH* potential of hydrogen, *HCO*_*3*_^*−*^ : ion bicarbonate

**Fig. 5:**
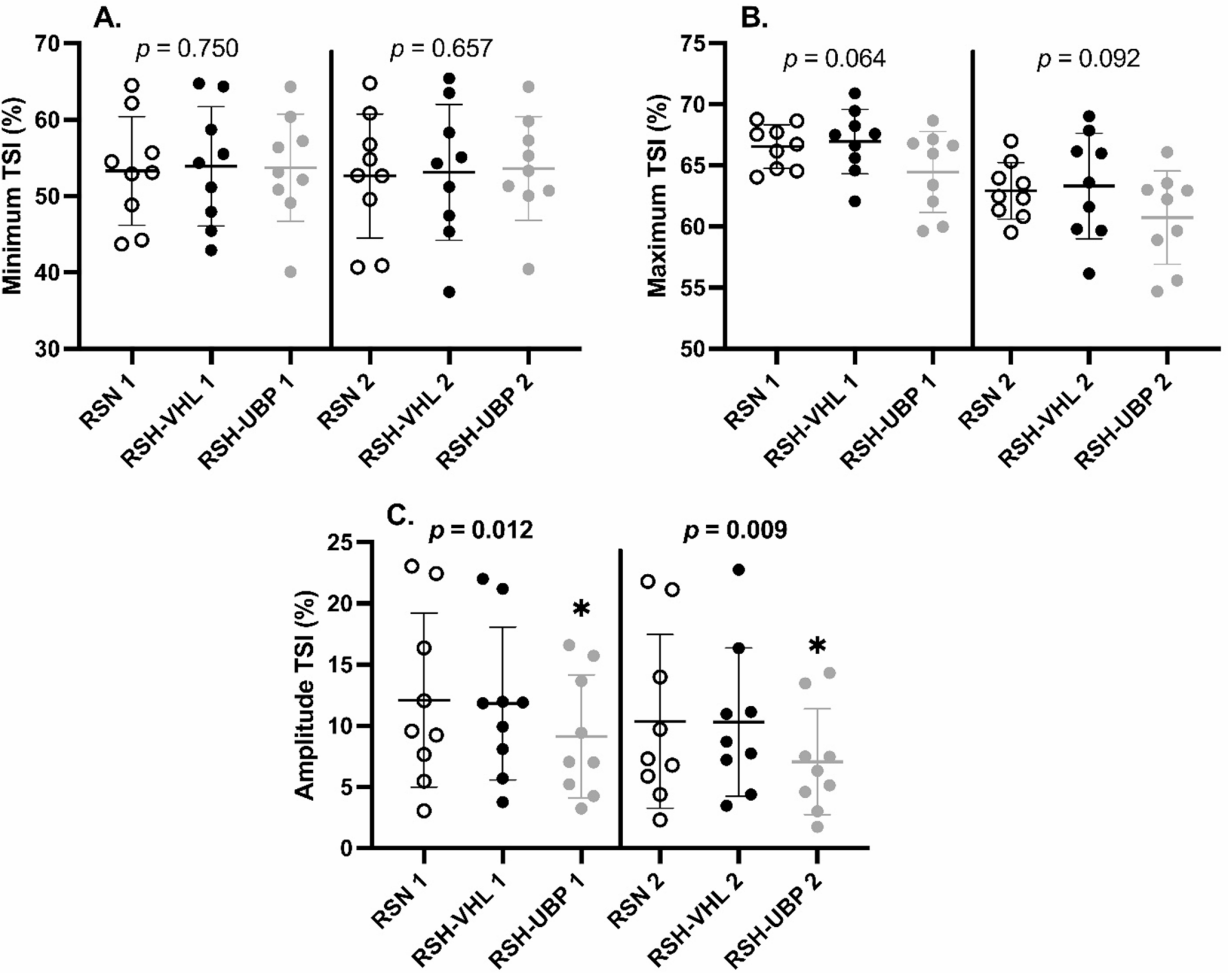
Tissue saturation index (TSI) in response to sprint session in unrestrictive breathing- (RSN), voluntary hypoventilation at low lung volume- (RSH-VHL), and end-expiratory breath-hold until the breaking point- (RSH-UBP) sessions. *Different from RSH-VHL

Muscle oxygenation data are presented in Table 2; Fig. 5. The minimum and maximum values of each NIRS index (O_2_Hb, HHb, THb, and TSI) did not differ significantly across the three sessions during the first set. However, there were significant differences in the amplitudes of ΔO_2_Hb (F = 3.748, *p* = 0.046), ΔTHb (F = 8.130, *p* = 0.004), and the amplitude of ΔTSI (F = 5.866, *p* = 0.012) during the first set. These differences persisted in the second set with significant differences in the amplitude of ΔO_2_Hb (F = 4.900, *p* = 0.022), ΔTHb (F = 4.683, *p* = 0.025), TSI (F = 6.518, *p* = 0.009). Post hoc comparisons with Bonferroni correction did not identify the specific conditions responsible for the difference in ΔO_2_Hb amplitude during the first set. For all other significant differences, the amplitude of the muscle deoxygenation-reoxygenation was lower in RSH-UBP than in RSH-VHL.

**Table 2:** Vastus lateralis oxygenation in response to sprint session in RSN, RSH-VHL, and RSH-UBP

Set	Variable	RSN	RSH-VHL	RSH-UBP	F statistic	*p*-value
First set	Minimum O_2_Hb (µM)	−13.2 ± 5.7	−15.7 ± 7.2	−14.7 ± 6.6	0.423	0.662
	Maximum O_2_Hb (µM)	−1.1 ± 6.9	−2.9 ± 6.9	−3.9 ± 5.0	0.298	0.292
	Amplitude ΔO_2_Hb (µM)	**10.1 ± 5.4**	**10.8 ± 4.9**	**8.3 ± 4.4**	**3.748**	**0.046**
	Minimum HHb (µM)	1.9 ± 2.7	2.1 ± 2.9	3.0 ± 4.4	0.309	0.738
	Maximum HHb (µM)	11.7 ± 6.6	11.7 ± 7.4	11.4 ± 8.4	0.032	0.905
	Amplitude ΔHHb (µM)	8.6 ± 6.0	8.5 ± 5.3	7.0 ± 4.0	2.282	0.134
	Minimum THb (µM)	−4.8 ± 4.4	−6.9 ± 8.8	6.2 ± 6.4	0.275	0.660
	Maximum THb (µM)	3.9 ± 7.2	2.3 ± 9.7	1.3 ± 8.0	0.397	0.586
	Amplitude ΔTHb (µM)	**7.6 ± 4.5**	**8.0 ± 3.8**	5.8 ± 3.0*	**8.130**	**0.004**
Second set	Minimum O_2_Hb (µM)	−12.3 ± 6.8	−14.8 ± 6.8	−14.6 ± 7.0	0.574	0.499
	Maximum O_2_Hb (µM)	−4.4 ± 7.1	−5.6 ± 5.9	−8.4 ± 5.3	0.986	0.395
	Amplitude ΔO_2_Hb (µM)	**8.0 ± 3.9**	**9.5 ± 4.1**	6.2 ± 3.2*	**4.900**	**0.022**
	Minimum HHb (µM)	5.4 ± 4.2	5.4 ± 4.1	5.9 ± 5.6	0.069	0.934
	Maximum HHb (µM)	12.2 ± 6.7	12.7 ± 7.8	11.4 ± 8.3	0.526	0.601
	Amplitude ΔHHb (µM)	6.8 ± 5.3	7.5 ± 4.9	5.4 ± 3.7	3.554	0.053
	Minimum THb (µM)	−2.7 ± 4.7	−4.6 ± 8.1	−5.6 ± 7.3	0.539	0.594
	Maximum THb (µM)	3.8 ± 6.0	2.5 ± 9.0	−0.4 ± 8.8	1.116	0.352
	Amplitude ΔTHb (µM)	**6.9 ± 3.3**	**7.3 ± 3.3**	5.3 ± 2.7*	**4.683**	**0.025**

*O*_*2*_*Hb* oxyhemoglobin, *HHb* deoxyhemoglobin, *THb* total hemoglobin. *p < 0.05 for difference with RSH-VHL

Cardiac hemodynamic data are presented in Table 3. SV and Q̇ were similar across the three conditions during both sets. Regarding HR, the only significant difference observed was in the HR total during the first set (K = 6.200, *p* = 0.045) with a lower HR in RSH-UBP compared to RSN.

**Table 3:** Cardiac hemodynamic responses to sprint session in RSN, RSH-VHL, and RSH-UBP

Set	Variable	RSN	RSH-VHL	RSH-UBP	Test statistic	*p*-value
First set	HR sprint (bpm)	156 ± 9	151 ± 11	149 ± 14	F = 2.932	0.079
	**HR recovery (bpm)**	**160 ± 10**	**155 ± 11**	**148 ± 16**	**K = 8.600**	**0.014**
	**HR total (bpm)**	**158 ± 10**	**154 ± 11**	**148 ± 15** ^**†**^	**K = 6.200**	**0.045**
	SV sprint (mL)	135 ± 20	126 ± 22	137 ± 14	F = 1.076	0.362
	SV recovery (mL)	133 ± 19	128 ± 24	138 ± 16	F = 0.534	0595
	SV total (mL)	134 ± 19	128 ± 23	137 ± 15	F = 0.673	0.522
	Q̇ sprint (L.min^−1^)	21.2 ± 3.2	19 ± 3.9	20.7 ± 2.5	F = 1.559	0.237
	Q̇ recovery (L.min^−1^)	21.4 ± 3.4	20.1 ± 4.4	20.8 ± 2.0	F = 0.639	0.539
	Q̇ total (L.min^−1^)	21.3 ± 3.3	19.8 ± 4.2	20.7 ± 2.2	F = 0.911	0.420
Second set	HR sprint (bpm)	153 ± 14	153 ± 10	151 ± 13	F = 0.296	0.747
	HR recovery (bpm)	155 ± 17	155 ± 9	154 ± 12	K = 0.800	0.670
	HR total (bpm)	154 ± 16	154 ± 10	153 ± 12	K = 1.400	0.497
	SV sprint (mL)	136 ± 25	133 ± 23	137 ± 15	F = 0.097	0.908
	SV recovery (mL)	131 ± 24	134 ± 23	136 ± 17	K = 0.200	0.905
	SV total (mL)	134 ± 25	133 ± 23	136 ± 16	F = 0.063	0.939
	Q̇ sprint (L.min^−1^)	21.2 ± 3.7	20.7 ± 4.3	20.6 ± 2.5	F = 0.106	0.900
	Q̇ recovery (L.min^−1^)	19.9 ± 4.8	21.1 ± 4.2	21.1 ± 3.2	F = 0.535	0.595
	Q̇ total (L.min^−1^)	20.8 ± 3.7	21.0 ± 4.2	20.9 ± 2.9	F = 0.006	0.994

*HR* heart rate, *SV* stroke volume, *Q̇* cardiac output. ^†^*p* < 0.05 for difference with RSN

## Discussion

This study investigated the acute physiological responses to RSN, RSH-VHL, and RSH-UBP. The main findings are as follows: (1) RSH-VHL and RSH-UBP elicited greater hypoxic and relative hypercapnic stimuli than RSN; (2) The training load was reduced during RSH-UBP compared to the other two conditions; (3) Cardiac hemodynamic responses were not significantly different among the three conditions; (4) The muscle deoxygenation-reoxygenation amplitudes were smaller during RSH-UBP than in RSN and RSH-VHL.

While the physiological responses to RSH are well-documented with more than 100 articles published in the last decade (Faiss et al. 2024), few studies have investigated end-expiratory breath-hold until the breaking point at 150% of maximal aerobic power (Woorons et al. 2021b), 100% to 60% of maximal aerobic speed (Woorons et al. 2021a), or 125% of maximal aerobic speed (Woorons et al. 2023). However, these studies did not examine UBP during all-out effort. Furthermore, this study is the first to compare two different patterns of hypoventilation during repeated sprint training. Consistent with our hypothesis, greater hypoxic (Fig. 2) and relative hypercapnic stimuli (Fig. 3A) were observed during RSH-UBP and RSH-VHL compared to RSN. However, no significant difference in hypoxic or relative hypercapnic stimuli was observed between RSH-UBP and RSH-VHL.

Moreover, based on the blood analysis (Table 1), acidosis levels did not differ between the three sessions. Increased acidosis, attributed to higher energy supply from anaerobic glycolysis, has been reported during submaximal exercise with VHL (Kume et al. 2016; Toubekis et al. 2017). However, anaerobic glycolysis generally contributes less significantly to total energy supply during repeated sprints (Girard et al. 2011), at least with such sprint duration and exercise-to-rest ratio (Raberin et al. 2022). During a single 6-s sprint, anaerobic glycolysis accounts for approximately 40% of total energy production, with its contribution decreasing as sprints are repeated (Girard et al. 2011); phosphocreatine resynthesis playing a more prominent role in energy supply (Girard et al. 2011). Another possible explanation is that the greater oxygen consumption during the RSH-VHL recovery periods may facilitate acidosis buffering in comparison to RSN, as previously suggested (Woorons et al. 2017, 2019b).

Regarding the effects of lactate level during repeated sprints in hypoxia vs. normoxia, existing data are equivocal: elevated (Bowtell et al. 2014), unchanged (Woorons et al. 2019a; Rosa et al. 2024) or even lower (Woorons et al. 2017) blood lactate levels have been reported, compared to RSN. Reduced acidosis, despite a greater hypoxic stimulus in RSH-VHL, has been attributed to either enhanced lactate oxidation or greater phosphocreatine resynthesis during rest periods between sprints. The latter may result from higher oxygen uptake following sprints performed under RSH-VHL compared to RSN (Woorons et al. 2017; Rosa et al. 2024).

An unexpected finding of the present study is the lack of difference in blood gas values between the two hypoventilation conditions and RSN. While the time spent below 96% and 94% SpO_2_, the minimum SpO_2_ and the P_ET_CO_2_ values differed, the pre- to post-sprint changes in PO_2_ and PCO_2_ were similar. This may arise from methodological limitations, i.e., the post-capillary blood sampling required a few seconds to complete after the last sprint. During this time, participants experienced intense hyperventilation, which may explain the discrepancy between the SpO_2_ and P_ET_CO_2_ values monitored during the sprint session and the blood gas measurements. This raises questions about the most appropriate method to monitor hypoxic and relative hypercapnic stress during RSH-VHL sessions. Further research comparing continuous P_a_O_2_ and P_a_CO_2_ (with catheter), P_ET_CO_2_ and SpO_2_ would help to clarify this point.

While the hypoxic and relative hypercapnic stimuli did not differ between RSH-VHL and RSH-UBP, the training load during RSH-UBP was largely compromised compared to the other two conditions (Fig. 3E). Although mean power during the second set was higher in RSH-UBP than in RSH-VHL, the reduced sprint duration in RSH-UBP resulted in lower total work over the two sets. Specifically, the cumulative sprint durations in RSH-UBP were 55.4 ± 12.7 and 53.0 ± 10.3 s for the first and second set, respectively; significantly lower than the fixed 80-s (8 × 10-s) of sprinting in RSH-VHL and RSN. This is due to the difficulty of maintaining sprints longer than 6–7 s with an end-expiratory breath-hold. Another explanation is the mental strain induced by apnea (Elia and Lemaître 2025). Given that RSH-UBP may impose more challenging apnea duration than RSH-VHL, it is reasonable to hypothesize a higher level of mental strain in this condition.

To date, RSH-VHL has typically been performed with short sprint durations of 6 s (Woorons et al. 2017, 2019a; b; Lapointe et al. 2020; Rosa et al. 2024) or over short distances, such as 30 m (Ait Ali Braham et al. 2024), 40 m (Fornasier-Santos et al. 2018; Brocherie et al. 2023) in running, or 15 m in swimming (Trincat et al. 2017). However, these durations and distances may not align with the optimal sprint duration for different activities. Sprint duration, along with the exercise-to-rest ratio, has been shown to be critical for the effectiveness of RSH (Millet and Faiss 2012; Raberin et al. 2022, 2023).

Recent studies have reported longer exercise durations with UBP; e.g., 9.6 ± 0.9 s at 150% of maximal aerobic power (Woorons et al. 2021b); 10.1 ± 1.0 s at 125% of maximal aerobic speed (Woorons et al. 2023); 12.2 ± 1.7 s at 100% of maximal aerobic speed (Woorons et al. 2021a); 13.2 ± 1.8 s at 80% of maximal aerobic speed (21); and 10.1 ± 1.1 s at 60% of maximal aerobic speed (Woorons et al. 2021a). However, none of these studies examined all-out efforts. Interestingly, Woorons et al. (Woorons et al. 2021a) who conducted exercise with UBP at 60%, 80%, or 100% of maximal aerobic speed, did not report any correlation between exercise intensity and mean end-expiratory breath-hold duration.

A recent study implementing RSH-UBP in elite judo athletes observed a mean sprint duration of 9.4 ± 0.7 s across 233 ± 14 sprints during a 4-week training period (Woorons et al. 2024). This was significantly shorter than the 10-s sprint duration observed in the RSN group of the same study (Woorons et al. 2024). Differences in sprint duration with the current study could be attributed to several factors. Firstly, the populations were different, i.e., recreational athletes versus elite judo athletes. Judo athletes are likely accustomed to tolerating hypoxia due to the intermittent nature of their sport, which includes strangulation techniques. Secondly, the judo athletes likely improved their apnea tolerance over the 8-week training period due to both, learning effect and acclimatization. Thirdly, RSH-UBP was performed on a rowing ergometer, which involves a muscle relaxation phase during the handle return. This phase, roughly equal in duration to the contraction phase, allows partial recovery within each sprint cycle. This likely contributed to the participants ability to prolonged efforts.

Taken together, our findings show that maintaining breath hold longer than 6–7 s is difficult for inexperienced athletes, despite familiarization, as indicated by the high individual variability and the high minimum SpO₂ values reached, particularly during the first set. This lower training load could compromise training adaptations, reducing the effectiveness of the session and delaying overall progress. This may be a major limitation of RSH-UBP, but further research is needed to explore the feasibility of longer sprints, or longer sets, under RSH-UBP conditions.

Additionally, it is worth noting that in the present study, hypoxic and relative hypercapnic stimuli did not differ between RSH-VHL and RSH-UBP, despite the shorter sprint duration in the latter condition.

Regarding microvascular and muscular responses, our results demonstrated lower amplitudes in muscle deoxygenation-reoxygenation in RSH-UBP than in the other two conditions (Table 3; Fig. 5). In the absence of occlusion, changes in ΔTHb arise from alterations in oxygenated blood flow, such as changes in vasodilation or capillary recruitment, and are accompanied by corresponding changes in muscle oxygenation. Previous studies have reported lower ΔTHb during UBP compared to exercise with unrestricted breathing, potentially due to peripheral vasoconstriction redistributing blood toward the brain to mitigate severe hypoxemia (Woorons et al. 2021a). In the present study, while the hypoxic stimulus was similar between RSH-VHL and RSH-UBP, blood volume was lower in the latter condition. Therefore, the hypoxic stimulus is unlikely to explain per se the observed differences in ΔTHb.

The shorter sprint duration in RSH-UBP likely accounts too. With shorter sprints, the oxygen demand and the muscle intravascular occlusion (Smith and Billaut 2010; Faiss et al. 2013) may be time-limited, leading to reduced muscle deoxygenation-reoxygenation. Previous studies have reported differences in SpO_2_ during hypoventilation training without corresponding changes in muscle oxygenation (Woorons et al. 2019a; Imai et al. 2022). It has been hypothesized that participants were unable to enhance muscle O_2_ extraction sufficiently to compensate for the reduced O_2_ supply under RSH-VHL.

One proposed benefit of exercise with end-expiratory breath-hold until the breaking point is the significant increase in stroke volume, which consequently enhances cardiac output (Woorons et al. 2011; Convertino 2019). This phenomenon may result from the large and brief inspiration that occurs immediately after apnea, creating a “pump effect,” characterized by a substantial increase in venous return to the heart (Woorons et al. 2011). It is well-established that the negative intrathoracic pressure generated during inspiration enhances venous return, thereby increasing cardiac preload, SV, and Q̇ (Convertino 2019). Consequently, VHL, with or without maximal breath old, was suspected to amplify this mechanism. For instance, cycling at 65% of maximal power output in VHL induced increases in heart rate (HR), SV, and Q̇ compared to normal breathing (Woorons et al. 2011). Moreover, it has been hypothesized that exercise in VHL could promote cardiac adaptations that enhance SV (Woorons et al. 2019b, 2021b). However, only one study has investigated cardiac hemodynamics during VHL performed until the breaking point at high intensity (150% of maximal power output) (Woorons et al. 2021b). In this later study, a higher SV was observed at the end of the exercise bout and the beginning of recovery (Woorons et al. 2021b). This increase in SV was attributed to a greater change in intrathoracic pressure, which enhanced venous return, hypercapnic-related adrenergic stimulation, and a lower HR, allowing for greater ventricular filling. In the present study, SV did not differ across the three conditions. The RSH-VHL condition, which allows one ventilatory maneuver during the sprint period and therefore greater intrathoracic pressure changes, did not result in a higher SV, calling into question the previously proposed mechanisms. While impedance cardiography has been validated for use during maximal exercise (Richard et al. 2001) and shows good reliability during supramaximal efforts (Astorino et al. 2015), the exaggerated respiratory movements associated with all-out efforts and specific ventilatory maneuvers could potentially compromise the accuracy of these measurements and explain this discrepancy.

In the present study, average HR was also lower during the first set of RSH-UBP compared to the two other conditions. While the decrease in HR during breath holding could be attributed to an oxygen-conserving response aiming to reduce the cardiac work (Lindholm et al. 1999), the absence of a difference in HR between RSH-UBP and RSH-VHL suggests that the lower HR in RSH-UBP may primarily result from the reduced total work performed during this condition. Overall, our findings indicate that the physiological differences observed during RSH-UBP may be attributable to the shorter sprint duration.

## Limitations

First, regardless of the breathing modality used during the session (RSH-VHL or RSH-UBP), full cooperation and self-regulation by participants is essential. The amount and frequency of familiarization may influence participant engagement and the effective execution of the breathing modality. Second, while an exercise-to-rest ratio of 1:2, commonly considered effective for RSH, was used in this study, this ratio may affect sprint duration in the RSH-UBP condition, particularly when recovery periods fall below 15 s. To the best of our knowledge, the relationship between sprint duration under VHL, intensity, and exercise-to-rest ratio has not yet been systematically investigated. Various exercise-to-rest ratios have been applied in previous studies, including higher ratios (e.g., 1:1.5) at lower intensities (Woorons et al. 2021a), but this remains an open question that warrants further clarification.

From a practical training perspective, the relevance of our findings remains limited. While the present study provides physiological insights into the physiological responses to RSH-VHL and RSH-UBP, it does not directly assess whether these modalities improve performance or competitive outcomes. Controlled interventional studies are required to establish the transferability of these acute responses to real-world training benefits.

## Conclusion

Although RSH-UBP elicited greater hypoxic and relative hypercapnic stimuli than RSN, no significant differences were observed when compared to RSH-VHL. However, the training load was reduced in the RSH-UBP condition, likely due to participants inability to sustain prolonged apneas, which resulted in shorter sprint durations. As a result, muscle deoxygenation levels during RSH-UBP appeared diminished.

Future interventional training studies are needed to directly compare these two strategies and determine whether RSH-VHL or RSH-UBP is more effective for promoting consistent physiological adaptations without compromising training loads.

## References

[CR1] Ait Ali Braham M, Ouchen Y, Woorons X (2024) Effects of a 6-week repeated-sprint training with voluntary hypoventilation at low and high lung volume on repeated-sprint ability in female soccer players. Int J Sports Physiol Perform 19:463–470. 10.1123/ijspp.2023-0392 38412852 10.1123/ijspp.2023-0392

[CR2] Astorino TA, Bovee C, DeBoe A (2015) Estimating hemodynamic responses to the wingate test using thoracic impedance. J Sports Sci Med 14:834–840 26664281 PMC4657427

[CR3] Bishop D, Girard O, Mendez-Villanueva A (2011) Repeated-sprint ability - part II: recommendations for training. Sports Med 41:741–756. 10.2165/11590560-000000000-00000 21846163 10.2165/11590560-000000000-00000

[CR4] Bowtell JL, Cooke K, Turner R et al (2014) Acute physiological and performance responses to repeated sprints in varying degrees of hypoxia. J Sci Med Sport 17:399–403. 10.1016/j.jsams.2013.05.016 23809839 10.1016/j.jsams.2013.05.016

[CR5] Brocherie F, Girard O, Faiss R, Millet GP (2017) Effects of repeated-sprint training in hypoxia on sea-level performance: a meta-analysis. Sports Med 47:1651–1660. 10.1007/s40279-017-0685-3 28194720 10.1007/s40279-017-0685-3

[CR6] Brocherie G, Millet GP, Woorons X (2023) Effects of repeated-sprint training in hypoxia induced by voluntary hypoventilation on performance during ice hockey off-season. Int J Sports Sci Coaching 18:446–452. 10.1177/17479541221079531

[CR7] Casey DP, Joyner MJ (2012) Compensatory vasodilatation during hypoxic exercise: mechanisms responsible for matching oxygen supply to demand. J Physiol 590:6321–6326. 10.1113/jphysiol.2012.242396 22988134 10.1113/jphysiol.2012.242396 PMC3533194

[CR8] Casey DP, Madery BD, Curry TB et al (2010) Nitric oxide contributes to the augmented vasodilatation during hypoxic exercise. J Physiol 588:373–385. 10.1113/jphysiol.2009.180489 19948661 10.1113/jphysiol.2009.180489 PMC2821731

[CR9] Convertino VA (2019) Mechanisms of inspiration that modulate cardiovascular control: the other side of breathing. J Appl Physiol 127(5):1187–1196. 10.1152/japplphysiol.00050.2019 31225967 10.1152/japplphysiol.00050.2019

[CR10] Elia A, Lemaître F (2025) The application of breath-holding in sports: physiological effects, challenges, and future directions. Eur J Appl Physiol. 10.1007/s00421-025-05752-y 40126615 10.1007/s00421-025-05752-y PMC12354527

[CR11] Faiss R, Léger B, Vesin J-M et al (2013) Significant molecular and systemic adaptations after repeated sprint training in hypoxia. PLoS One 8:e56522. 10.1371/journal.pone.0056522 23437154 10.1371/journal.pone.0056522 PMC3577885

[CR12] Faiss R, Raberin A, Brocherie F, Millet GP (2024) Repeated-sprint training in hypoxia: a review with 10 years of perspective. J Sports Sci. 10.1080/02640414.2024.2416821 39445500 10.1080/02640414.2024.2416821

[CR13] Fornasier-Santos C, Millet GP, Woorons X (2018) Repeated-sprint training in hypoxia induced by voluntary hypoventilation improves running repeated-sprint ability in rugby players. Eur J Sport Sci 18:504–512. 10.1080/17461391.2018.1431312 29400616 10.1080/17461391.2018.1431312

[CR14] Girard O, Mendez-Villanueva A, Bishop D (2011) Repeated-sprint ability - part I: factors contributing to fatigue. Sports Med 41:673–694. 10.2165/11590550-000000000-00000 21780851 10.2165/11590550-000000000-00000

[CR15] Imai A, Yamaguchi K, Goto K (2022) Comparison of systemic and peripheral responses during high-intensity interval exercise under voluntary hypoventilation vs. hypoxic conditions. Phys Act Nutr 26:008–016. 10.20463/pan.2022.0008 10.20463/pan.2022.0008 PMC9395251 35982624

[CR16] Kume D, Akahoshi S, Yamagata T et al (2016) Does voluntary hypoventilation during exercise impact EMG activity? SpringerPlus 5:149. 10.1186/s40064-016-1845-x 27026846 10.1186/s40064-016-1845-x PMC4766162

[CR17] Lapointe J, Paradis-Deschênes P, Woorons X et al (2020) Impact of hypoventilation training on muscle oxygenation, myoelectrical changes, systemic [K+], and repeated-sprint ability in basketball players. Front Sports Act Living 2:29. 10.3389/fspor.2020.00029 33345021 10.3389/fspor.2020.00029 PMC7739750

[CR18] Lindholm P, Sundblad P, Linnarsson D (1999) Oxygen-conserving effects of apnea in exercising men. J Appl Physiol (1985) 87:2122–2127. 10.1152/jappl.1999.87.6.2122 10601158 10.1152/jappl.1999.87.6.2122

[CR19] Millet GP, Faiss R (2012) Hypoxic conditions and exercise-to-rest ratio are likely paramount. Sports Med 42:1081–1083. 10.1007/BF03262313 23106429 10.1007/BF03262313

[CR20] Millet G, Girard O, Beard A, Brocherie F (2019) Repeated sprint training in hypoxia – an innovative method. Dtsch Z Sportmed 2019:115–122. 10.5960/dzsm.2019.374

[CR21] Nava RC, McKenna Z, Fennel Z et al (2022) Repeated sprint exercise in hypoxia stimulates HIF-1-dependent gene expression in skeletal muscle. Eur J Appl Physiol. 10.1007/s00421-022-04909-3 35190865 10.1007/s00421-022-04909-3

[CR22] Perrey S, Ferrari M (2018) Muscle oximetry in sports science: a systematic review. Sports Med 48:597–616. 10.1007/s40279-017-0820-1 29177977 10.1007/s40279-017-0820-1

[CR23] Raberin A, Elmer J, Willis SJ et al (2022) The oxidative-glycolytic balance influenced by sprint duration is key during repeated sprint in hypoxia. Med Sci Sports Exerc. 10.1249/MSS.0000000000003042 36136604 10.1249/MSS.0000000000003042

[CR24] Raberin A, Willis SJ, Richard T et al (2023) Hypoxia does not change performance and psychophysiological responses during repeated cycling sprints to exhaustion with short exercise-to-rest ratio. Int J Sports Physiol Perform 18:213–217. 10.1123/ijspp.2022-0234 36640773 10.1123/ijspp.2022-0234

[CR25] Richard R, Lonsdorfer-Wolf E, Charloux A et al (2001) Non-invasive cardiac output evaluation during a maximal progressive exercise test, using a new impedance cardiograph device. Eur J Appl Physiol 85:202–207. 10.1007/s004210100458 11560071 10.1007/s004210100458

[CR26] Rosa CH, Monteiro CP, Barata C et al (2024) Cardiorespiratory and muscle oxygenation responses to voluntary hypoventilation at low lung volume in upper body repeated sprints. Eur J Appl Physiol. 10.1007/s00421-024-05569-1 39138688 10.1007/s00421-024-05569-1 PMC11568980

[CR27] Smith KJ, Billaut F (2010) Influence of cerebral and muscle oxygenation on repeated-sprint ability. Eur J Appl Physiol 109:989–999. 10.1007/s00421-010-1444-4 20354718 10.1007/s00421-010-1444-4

[CR28] Toubekis AG, Beidaris N, Botonis PG, Koskolou M (2017) Severe hypoxemia induced by prolonged expiration and reduced frequency breathing during submaximal swimming. J Sports Sci 35:1025–1033. 10.1080/02640414.2016.1209304 27431779 10.1080/02640414.2016.1209304

[CR29] Trincat L, Woorons X, Millet GP (2017) Repeated-sprint training in hypoxia induced by voluntary hypoventilation in swimming. Int J Sports Physiol Perform 12:329–335. 10.1123/ijspp.2015-0674 27294771 10.1123/ijspp.2015-0674

[CR30] Woorons X, Bourdillon N, Vandewalle H et al (2010) Exercise with hypoventilation induces lower muscle oxygenation and higher blood lactate concentration: role of hypoxia and hypercapnia. Eur J Appl Physiol 110:367–377. 10.1007/s00421-010-1512-9 20503056 10.1007/s00421-010-1512-9

[CR31] Woorons X, Bourdillon N, Lamberto C et al (2011) Cardiovascular responses during hypoventilation at exercise. Int J Sports Med 32:438–445. 10.1055/s-0031-1271788 21563023 10.1055/s-0031-1271788

[CR32] Woorons X, Mucci P, Aucouturier J et al (2017) Acute effects of repeated cycling sprints in hypoxia induced by voluntary hypoventilation. Eur J Appl Physiol 117:2433–2443. 10.1007/s00421-017-3729-3 29032393 10.1007/s00421-017-3729-3

[CR33] Woorons X, Dupuy O, Mucci P et al (2019a) Cerebral and muscle oxygenation during repeated shuttle run sprints with hypoventilation. Int J Sports Med 40:376–384. 10.1055/a-0836-9011 30900226 10.1055/a-0836-9011

[CR34] Woorons X, Millet GP, Mucci P (2019b) Physiological adaptations to repeated sprint training in hypoxia induced by voluntary hypoventilation at low lung volume. Eur J Appl Physiol 119:1959–1970. 10.1007/s00421-019-04184-9 31286240 10.1007/s00421-019-04184-9

[CR35] Woorons X, Billaut F, Vandewalle H (2020) Transferable benefits of cycle hypoventilation training for run-based performance in team-sport athletes. Int J Sports Physiol Perform 15:1103–1108. 10.1123/ijspp.2019-0583 32106076 10.1123/ijspp.2019-0583

[CR36] Woorons X, Billaut F, Lamberto C (2021a) Running exercise with end-expiratory breath holding up to the breaking point induces large and early fall in muscle oxygenation. Eur J Appl Physiol 121:3515–3525. 10.1007/s00421-021-04813-2 34532775 10.1007/s00421-021-04813-2

[CR37] Woorons X, Lemaitre F, Claessen G et al (2021b) Exercise with end-expiratory breath holding induces large increase in stroke volume. Int J Sports Med 42:56–65. 10.1055/a-1179-6093 32842157 10.1055/a-1179-6093

[CR38] Woorons X, Daussin F, Combes A, Mucci P (2023) Physiological responses to supramaximal running exercise with End-Expiratory breath holding up to the breaking point. J Hum Kinetics 90:111–123. 10.5114/jhk/174465 10.5114/jhk/174465 PMC10875693 38380296

[CR39] Woorons X, Faucher C, Dufour SP et al (2024) Hypoventilation training including maximal end-expiratory breath holding improves the ability to repeat high-intensity efforts in elite judo athletes. Front Physiol. 10.3389/fphys.2024.1441696 39397858 10.3389/fphys.2024.1441696 PMC11467534

